# A New Field of Practice

**Published:** 1914-11

**Authors:** T. D. Crothers

**Affiliations:** Hartford, Conn.


### A New Field of Practice.

By T. D. CROTHERS, M. D., Hartford, Conn.

The advent of the Gold Cures startled public opinion, which had been slumbering along for years, in the conviction that drinking spirits was a mere wilful weakness and depended upon the low morals and ethical standards of the individual. The theories that it was a physical disease, and curable as others are, was treated with a sort of a silent contempt as most unreasonable and unworthy of consideration, and the small number of people who advocated such theories were the extremists and dreamers of the age.

The Gold Cure advent and the enthusiastic endorsements and pretensions made for it by the persons who claimed to have been thoroughly cured, startled the public, and even physicians who denounced the secrecy and pretension, were willing to acknowledge that there was some value in it. A great wave of new sentiment sprung up, especially among persons who drank, and with it came a desire to be helped and use the means that promised so much, and that was followed by a new sentiment that the drink evil was a curable one, and in this way, the quacks and pretenders have been the fore-runners of a new conception of the possibilities of curing the drink neuroses.

This is a repetition of history and the quack age of every advance of civilization, and discovery. Whenever a new step forward is taken, empirics are ready to utilize it for personal advantage in the most extravagant way. By and by a larger knowledge of the subject reveals the failure of the quacks and the weakness of their theories, and brings out the real truths and the real workers go on developing and cultivating the lands which have been discovered.

The empirical cures by secret drugs and secret methods are passing away, but they have educated the public, they have broken up the old theories and revealed a new world of practice. The work in the laboratories has brought ample confirmation in the testing of the effects of alcohol on cell and tissue, showing its anesthetic action, and breaking up a whole world of theories that have been held as sacred as the Bible.

All this has given new impetus to temperance reformers and anti-alcoholic literature, and more than this, it has called attention to the direct and indirect injuries resulting from the use of spirits, and this has opened another field, which today puts alcohol down as one of the most prominent causes of disease, next to syphilis.

Clinical students are coming to recognize these facts and already a considerable literature has sprung up, showing how alcohol acts on the cell and tissue, and to what degree it intensifies the progress of disease and diminishes vital resources. The facts which I have, for over forty years, been pressing on public attention, that alcoholism is a neuroses and psychosis, and can be traced, understood and treated the same as epilepsy, or any form of insanity, has been endorsed and is now held by a great many of the leading students of mental disease.

In the field of therapeutics, the quack has been prominent in the promotion of drugs and drug combinations, but these have not been confirmed by exact studies, however a great many drugs have been found very useful in diminishing and breaking up the toxemiac effects from spirits, and lessening the anemias and convulsive neuroses which were active causes of these conditions. In company with others, I have pointed out a great variety of means and measures, that when applied properly are followed by the most gratifying results.

It is possible to say at present, that the drink and drug neurotics is positively curable in a general way, by means and measures suitable to each person and the particular conditions which they present. In the fifty years, since the advent of Binghamton Asylum, which attracted hundreds of inebriates, there has been an increasing number of inebriates and alcoholics coming to medical men for help. A very large proportion of these were chronic cases who sought relief during the drink paroxysms, and after they had subsided, considered themselves cured and gave little or no attention to medical means for farther relief.

During the last few years this belief is rapidly passing away. While there are a great many men and women going to hospitals and homes for relief from the drink paroxysm, the idea is growing that this should be prevented, and not allowed to culminate in acute intoxication. In the commercial world and in the professional circles, the moderate or excessive drinker is looked upon with more and more suspicion every year.

In positions of trust and responsibility he is recognized as incapable and more or less inefficient, and likely to peril his own, as well as other's interests, by faults of brain and nerve power. Insurance societies find from actual demonstrations that the moderate drinker has a higher mortality and greater liability to accidents. The bonding societies find that there is greater loss of property through errors and defalcations and mistakes from this class.

Thus everywhere a fear of disaster, loss and disability is growing in public opinion in the work of persons who use spirits and drugs. Hence such persons are being restricted, and turned away from responsible positions through fear of their failures. This is very evident in the increasing number of persons who call themselves moderate drinkers, who are now seeking means for relief. They are very careful to avoid Gold Cure establishments for fear of loss of reputation, but with the aid of their physicians, they claim a great many kinds of nervous disorders and neuroses, diseases of the heart and liver, and go to sanatoriums, and water cures for relief. At one of the favorite springs in the West it was estimated that over 90% of all the patients who were under treatment, were literally inebriates, or so-called moderate drinkers, and yet they came for all sorts of disabilities, and posed as victims of over-work and other disorders.

In my own experience an increasing number of persons come by advice of the physician for so-called insomnia, exhaustion, disturbed nutrition, and nerve derangements. The fact of having used spirits was considered of minor importance. In reality it is the active exciting cause, and when removed recovery follows rapidly. These people would repel the idea of being called inebriates, and yet to their friends and family physician, they are so perilously near this condition, as to call for special treatment.

In business and professional circles, there is an increasing pressure brought to bear on all persons who use spirits, and show some kind of decline, and their failure to go under treatment at once, and not wait for more disastrous conditions, is fatal.

This is the trend of modern science, and this is supported by practical experience as well as theoretical studies. The man or woman who boasts of ability to drink moderately in positions of trust and responsibility, realizes by the pressure of friends and public interest that he is regarded with suspicion, and this is deepening every day, and calling for correction. By and by it dawns on him that he must make an effort to correct this or conceal his private habits from his friends.

The physician very quickly discovers defects of organism and suggests a short treatment in some hospital or sanatorium. Here he learns the true condition and comes to recognize the rising tide of public sentiment that calls for only the most vigorous men and women who are abstainers in the strictest sense, to do the work of the world.

He learns from the specialist, what he must do, and how he must do it. From this there is a new field of medical help opened. Should he relapse and drink again, the question is now to recover, not by quack means and methods, but from expert help, and something that is real and tangible. He makes a confidant of his family physician, and comes to rely upon him, and in this way he is prevented from falling into the most disastrous consequences, losing his reputation and business, and dying from intercurrent disease.

A very prominent banker made this statement. "I have drank moderately at dinners and now and then, on other occasions, when I discovered that I was not regarded as formerly. My associates did not take my word as implicitly as before. They suggested to me, that I was over-worked and needed a rest. Finally, it dawned on me, that it was my alcohol breath and club life more than anything else. I went to a hospital and told the physician that there was something wrong about me."

After a time he frankly told me, that the fault was wine and spirits; that if I would abstain from these, I would soon regain my health. I did so, and since that time I have been very urgent to all my friends and associates to stop the use of spirits and save themselves from innumerable physical and mental troubles, and wherever my advice has been accepted the best results have followed.

This statement very clearly indicates a new field of actual practice, not for the use of drugs, but mental and physical means that will react at once.

In my experience of over forty years in the care and treatment of spirit and drug takers, I have found that thoughtful men and women in all circles of society who have found themselves obliged to use spirits and drugs for temporary relief, and making more and more efforts to recover and to practically escape from the extreme effects which are certain to follow. Many persons occupying responsible positions are encouraged by their family physicians to go away, and take a rest, when practically they need abstinence from spirits, more than anything else.

There is in the strain and stress of modern civilization, an increasing number of men who at times give way to alcoholic crazes and go at once to some hospital or sanatorium for relief. After a short time the paroxysm passes off and they resume business again. The family physician is painfully conscious of his inability to treat them at home, because they want change of surroundings, isolation and personal attention and care from some competent source. It cannot be had from drugs alone, but must come from change and persistent effort to become restored. Many hospitals and institutions receive people in the acute stages, and after various courses of treatment, send them home restored. This is a great work, but a greater field beyond this is dawning where men at the beginning or at the first onset of the drink neuroses, are persuaded to go and learn the methods of restoration.

The moderate drinker who is aware that his use of spirits is impairing his vigor, or if not personally recognizing this, his friends recognize it for him, and urge that he take a rest, and if that rest is in a proper sanatorium, he realizes for the first time, the dangerous ground that he is on, and makes an effort to stop.

The paroxysmal drinkers, of which there is a great army growing constantly larger, who at times is impelled to use spirits or drugs to relieve the tension, needs something more than his family physician's prescription. He needs to go to an institution, where he can have nerve rest, and nerve treatment, until restoration follows. That's the new field that is opening for sanatoriums. When they provide hydropathic, electrical and other modern methods for recovery, they are veritable meccas for large numbers of men who otherwise would be lost. There is no sentiment in this. The facts are already accumulated to show that if we can help the moderate drinker, and restore the spasmodic drinker, we are checking a host of diseases at the fountain head, along lines of applied science.

The family physician who has the confidence of the patient, ought to direct his patrons to institutions to begin restorative processes, and then on their return continue the means and measures that will enable them to escape from an evil which in many instances is of greater magnitude than syphilis, and when combined with specific diseases is almost certain to break out in the most disastrous way in the future.

Whatever theories one may entertain, he must recognize the anaesthetic effect of alcohol, and its toxemiac action. The demand for it is a neuroses, possibly inherited, always acquired, and the results are exceedingly uncertain. This is the field for a new psychoses, a new therapeutics and a new practical application of modern means and measures.

---

**Endobronchial Treatment of Asthma.** Guisez, Bull, et Mem. de la Soc. de Med. de Paris, May 8, 1914, holds that asthma is not a neurosis, that in every case examined with the bronchoscope, there is bronchitis of the medium and small sized branches of the bronchi, with red, thickened, and vascularized mucosa. Instead of using sprays through the bronchial catheter and attempting to have the patient insufflate them, according to the method of Ephraim, the author merely deposits 20-25 c.c. of medicament into each bronchus with a slightly curved canula. The procedure is as follows: 1. Anaesthesia of the glottis and sub-glottis by "plastering" with 5% cocaine; 2. Injection into the trachea and the origin of the large bronchi of 3 or 4 c.c. of 5% alipin, with one or two drops of adrenalin. Wait 4 or 5 minutes. Injection of alipin, having the patient lie on the side of the bronchus to be injected. 5. Injection of "huile gomenelee faible" 5%.

